# Engaging and staying engaged: a phenomenological study of barriers to equitable access to mental healthcare for people with severe mental disorders in a rural African setting

**DOI:** 10.1186/s12939-017-0657-0

**Published:** 2017-08-29

**Authors:** Maji Hailemariam, Abebaw Fekadu, Martin Prince, Charlotte Hanlon

**Affiliations:** 1 Department of Psychiatry, College of Health Sciences, School of Medicine, Addis Ababa University, Addis Ababa, Ethiopia; 2 Center for Innovative Drug Development and Therapeutic Trials for Africa, Addis Ababa University, Addis Ababa, Ethiopia; 3 Institute of Psychiatry, Psychology and Neuroscience, Department of Psychological Medicine, Centre for Affective Disorders, King’s College London, London, UK; 4 Institute of Psychiatry, Psychology and Neuroscience, Health Services and Population Research Department, Centre for Global Mental Health, King’s College London, London, UK

**Keywords:** Poverty, Caregivers, Access, Task-sharing, Primary care, Sub-Saharan Africa, Ethiopia, Community mental health services, Mental health

## Abstract

**Background:**

In low-and middle-income countries, integration of mental health into primary care is recommended to reduce the treatment gap. In this study we explored barriers to initial and ongoing engagement of people with severe mental disorders (SMD) in rural Ethiopia after implementing integrated primary mental healthcare services.

**Methods:**

A qualitative approach was employed. In-depth interviews were conducted with 50 key informants: service users/caregivers engaged with care (*n* = 17), non-engagers and their caregivers (*n* = 10), those who had initiated treatment but disengaged and their caregivers (*n* = 12) and primary healthcare professionals (*n* = 11). Two focus group discussions were conducted with community health workers (10 per group). Thematic analysis was used.

**Results:**

Most respondents reported improved access to care, usually equated with medication, and were motivated to remain engaged due to experienced benefits of care. However, four main barriers to engagement emerged. (1) Poverty: resulting in inability to pay for medication and undermining vital social support affected engagement for all respondents. (2) Unreliable medication supplies and lack of second line options for inadequate response or intolerable side-effects. (3) The long-term nature of the illness: expectations of cure, stigma of chronic illness, low awareness about the illness and treatment and declining social support over time. (4) The nature of SMD: difficulty conveying the person when acutely disturbed and no flexibility for proactive outreach or legal frameworks to provide care when patients lacked capacity. In those who never engaged, geographical inaccessibility was an important barrier. Alternative cultural explanations for illness were only mentioned as a barrier only by two of the respondents.

**Conclusion:**

Economic interventions may be needed to support ongoing engagement in care for people with SMD. Systems of care for chronic illness need to be strengthened in combination with legal frameworks. Expanded options for affordable and effective medication and psychosocial interventions are required for person-centred care.

**Electronic supplementary material:**

The online version of this article (10.1186/s12939-017-0657-0) contains supplementary material, which is available to authorized users.

## Background

Supporting recovery in a person with a severe mental disorder (SMD; referring to psychotic and affective disorders associated with enduring disability) requires prompt and ongoing access to medical, psychological and social services. In high-income countries, one in three people with SMD who have accessed evidence-based mental healthcare drop out of care prematurely [1]. Discontinuation of mental healthcare in such settings has been associated with younger age, male gender, lack of insight, expectations of cure and the presence of co-morbid alcohol and substance use disorders [1]. In low-and middle-income countries (LMIC), initial access to mental healthcare is very limited, with close to 90% of cases never having contact with a biomedical care provider [2].

In LMICs, specialist mental healthcare providers and services are scarce. Thus, integration of mental healthcare into primary health care (PHC) through task sharing is a recommended approach to improve access to care [3, 4]. Training of non-specialist healthcare providers at primary care level can improve detection and initial management of selected mental and neurological disorders [5]. Integration of mental healthcare into primary care is also expected to promote long-term access and improve both mental and physical health outcomes for people with SMD [6]. However, there is little evidence from LMICs about the extent to which long-term engagement in care can be achieved and the factors associated with ongoing engagement or disengagement. In rural settings of LMICs [7] and high income countries with a high proportion of rural residence [8], evidence on access to mental healthcare relates to centralised and specialised mental health services.

Our previous qualitative study was conducted in a community where integrated primary care-based mental healthcare was about to be introduced. Respondents were selected from potential service users, caregivers, health professionals, traditional healers and healthcare administrators. The respondents anticipated that the existence of competing explanatory models, preference for traditional medicine and transport difficulties would be the biggest barriers to initiation and continuation of care [9]. In the study described in this paper, we explored the perspectives of people with SMD, their caregivers and health professionals on actual barriers to treatment initiation and continuing engagement with the primary care based mental healthcare which had been operating in the district for more than six months.

## Methods

### Study design

A phenomenological study design was followed to address the objectives. Phenomenological design allows study participants to describe their lived experiences regarding a particular phenomenon and draw meanings out of their everyday encounters [10, 11]. Phenomenological design also provides researchers with the flexibility to grasp the essence and structure of human experiences and understand how various complex meanings are constructed [12]. In this study, participants were asked about their interactions with PHC-based mental healthcare with a particular emphasis on their experiences with initiation and ongoing engagement with care.

### Study setting

The study was conducted in Sodo, a rural Ethiopian district in the Gurage zone located in the south central part, 100 km South of Addis Ababa. With a population of close to 180,000 people, Sodo district constitutes one of the most populous districts in the Gurage zone [13]. Agriculture is the main means of subsistence, adopted by 90% of the rural residents [13]. Ethiopian Orthodox Christianity constitutes the dominant religion of the district.

The baseline coverage of mental healthcare was reported to be very low with only 10% of people with schizophrenia having access to biomedical care [2]. In a study conducted in the neighbouring district, the lifetime prevalence of schizophrenia is reported to be 0.5% [2, 14]. The prevalence for bipolar disorder is 0.6% for males and 0.3% for females while major depression has 11% prevalence at the healthcare facility level [15, 16].

Sodo district is the implementation site for the PRogramme for Improving Mental health carE (PRIME) project [5]. PRIME works to develop evidence for the implementation and scale-up of integrated primary care-based mental healthcare for four priority mental and neurological disorders (SMDs, depression, epilepsy and alcohol use disorders) in five LMIC settings [17]. Since 2012, PRIME has provided brief training for Health Extension Workers (HEWs; group of community health workers with one year training in basic preventive care), community elders and project data collectors from the community to assist with detection and referral of people with a possible mental disorder. The HEWs were also trained to conduct community outreach to people with SMD who discontinued treatment to encourage them re-engage with care [5]. Facility-based primary care workers from seven government health centres were trained with the WHO’s mental health gap action programme (mhGAP) guideline. The health professionals involved in the intervention include clinical nurses, pharmacists and public health officers. The mhGAP training covered contents of diagnosis and management of selected priority disorders (SMD, depression, epilepsy and alcohol use disorders) at PHC level. Like many parts of the country, healthcare in Sodo district is financed by out-of-pocket expenditures. Only people who are considered as ‘poorest of the poor’ according to the sub-district administration may qualify for fee-waivers. A visit to the nearest primary mental health provider could cost up to 100 Ethiopian birr ($4.5). In a formative study conducted prior to the PRIME intervention, several religious healing sites were identified that are accessed by community members to manage mental and other health problems [18].

### Sampling

Selection of study participants was purposive [19]. Lists of all service users who had initiated primary care-based mental healthcare were obtained from each of the eight PHC facilities in Sodo district. Service providers at PHCs assisted in identification of people who had been consistent with their treatment and those who had missed two or more scheduled visits. The engaged and disengaged service users and their caregivers were then contacted for interview. The HEWs had copies of the referral slips used for all people identified within the community as having possible SMD. By cross-linking with the PHC records we were able to identify people with probable SMD who had never engaged with the primary care-based mental health service. Selected participants who were identified as non-engaged and their caregivers were invited for interview.

Facility-based health professionals who had been trained to provide mental healthcare were selected purposively for interview based on their level of involvement. Two focus group discussions (FGDs) were held with groups of HEWs who were selected from health posts of different sub-districts (*kebeles)* within the intervention site.

### Data collection

Data were collected between June 2015 and March 2016. Led by CH, qualitative data collection instruments were developed centrally for all PRIME countries. The topic guides were then adapted and translated for the Ethiopian intervention site. CH and MH reviewed the topic guides and conducted the contextual adaptation. The topic guides covered different domains of potential relevance as barriers and facilitators to access, including illness attribution, stigma and discrimination, social support, transport challenges and patterns of help-seeking.

The interviewer (MH, a social work professional with previous experience of conducting qualitative interviews in the study setting) carried out visits to the homes of study participants. To ensure reflexivity during the interviews, participants were told that there is no right or wrong answer and all we are interested in was their opinions. Enhanced probing techniques were used to avoid assumptions and premature conclusions about the data [20]. Whenever necessary, attempts were made to frame interviews as reciprocal conversations to maximise trust between the researchers and the study participants. Moreover, the first few transcripts were shared with one of the co-authors (CH), to suggest possible areas for more probing and discuss ways to strengthen data quality. In all cases, preferred interview locations were suggested by the study participants and privacy of the interview location was taken into account. In-depth interviews lasted between 45 and 90 min.

We had to opt for in-depth interviews with people with SMD and their caregivers for two main reasons. Each of the individuals we interviewed shared something sensitive which we think they wouldn’t have disclosed otherwise. Second, FGDs were not possible as most of these interviews were conducted in the houses of people. Bringing a large group was particularly difficult in this setting due to the lack of homogeneity of the participants, logistical challenges and difficulty of the terrain. However, we used two different ways to triangulate. (1) For methodological triangulation, we have supplemented the in-depth interviews with results from FGDs with the HEWs. Two PRIME staff members (women with social work and public health professional background and experience in qualitative research), facilitated the FGDs in an outpatient department of a health centre. Both FGDs lasted around 90 min. FGDs were possible with HEWs due to the relative accessibility and homogeneity of the group. (2) We conducted another quantitative study which also addressed issues of engagement with PHC-based mental healthcare.

### Data analysis

Interviews and FGDs were conducted in Amharic, the official language of Ethiopia. All study participants were assigned identification codes. Sociodemographic information linked to each identification code was kept in a password protected file separate from the interview transcripts. Audio files were renamed with the identification codes and names of people were omitted from all transcripts. Three women with experience in Amharic transcription were orientated to undertake the transcription and the whole process was supervised by MH. The interviews were translated to English by an experienced translator who is familiar with the study setting, with care taken to ensure semantic equivalence. Thematic analysis, a pragmatic technique for the management and synthesis of qualitative data, was utilised [21]. Two of the authors (MH and CH) carried out the coding process using OpenCode 4.02, an open-source qualitative data analysis software [22]. After coding the interviews independently, the coders swapped OpenCode files for cross-coding to establish deeper understanding of the codes. MH and CH held subsequent meetings to discuss the codes and emerging themes. A comprehensive codebook was developed and discussed by two of the authors (see Additional file 1). Field notes, essays and memos were used to supplement the interviews. In cases of disagreement, original Amharic transcripts were consulted to establish consistency. Patterns and relationships between and among themes were checked and different categories of respondents were compared.

## Results

A total of 88 people were invited to participate in the study, of whom 18 were not eligible due to not speaking Amharic (*n* = 5), being too unwell to be interviewed (*n* = 10) or refusal (*n* = 3). In-depth interviews were conducted with 50 people, including service users who were in treatment (*n* = 10), caregivers of people with SMD who were in treatment (*n* = 7), people with SMD who had never accessed care (*n* = 3) and caregivers (*n* = 7), people with SMD who had initiated treatment and then disengaged (*n* = 4) and their caregivers (*n* = 8). Additional interviews (*n* = 11) were carried out with health professionals at PHC facilities. The FGDs involved 20 HEWs (*n* = 10 each) in two separate groups. The sociodemographic details of the study participants are reported in Table 1.

**Table 1:** Sociodemographic characteristics of study participants

Participant type	Gender		Age (years)	Residence		Educational level		
	Male	Female		Urban	Rural	No formal education	Primary school only	Secondary and above
Caregivers of people with severe mental disorder, disengaged	1	7	28–70	2	6	6	1	-
People with severe mental disorder, disengaged	3	1	30–54	1	4	4	-	1
Caregivers of people with severe mental disorder, non-engaged	3	4	18–65	2	5	1	2	4
People with severe mental disorder, non-engaged	3	-	30–44	1	2	2	1	-
Caregivers of people with severe mental disorder, engaged	4	3	28–57	3	4	1	2	4
People with severe mental disorder, engaged	6	4	28–56	3	7	5	3	2
Health extension workers	-	20		-	20			20
PHC staff	11	-	25-	3	8	-	-	11

### Non-engagement, engagement and disengagement

Most respondents reported improved access to care and were motivated to remain engaged due to their experience of benefitting from the available treatment.

*It [my health] improved after the treatment. If it wasn’t for the tablets, I wouldn’t have been able to function. I would have entered into fights with people when they say nasty things about me. The medication adds patience to people.* IV20, female service user, engaged.

Compared to those who had engaged, non-engagers spoke more of differing cultural beliefs, although non-acceptability of biomedical care was only mentioned by two respondents. Some of the non-engagers endorsed geographical distance as a barrier:

*I live about four hours walk away from the health centre. I can’t walk fast when I had to bring him [the service user] to the health centre. He gets tired and wants to rest at different spots on our way. I have to sit down for him to rest and be able to walk again. There is no other transportation option to the health centre.* IV26, female caregiver, engaged.

However, the predominant barriers to care for most of those who had never engaged were the same as those who had disengaged and even for those who were engaged currently with treatment.

Four major sets of barriers to continuous engagement emerged: (1) Poverty (2) medication (3) long-term care and (4) the nature of SMD.

### Poverty

Poverty was the most frequently endorsed reason for inability to initiate treatment or maintain ongoing access to mental healthcare at PHCs. Reference to poverty was made by participants in all groups, even if they were still engaged with care.

*I have come across people who would say they cannot afford medications unless someone else pays for them. Some people returned to their homes with their prescription paper without buying the medication.* PHC staff, ID03.

Some of the PHC staff members said that they feel obliged to cover treatment costs for destitute people who are unable to pay for themselves. Although a fee waiver is available for the ‘poorest of the poor’, the service users and caregivers reported that the process is lengthy, and eligibility criteria are sometimes imprecise or applied inconsistently.

*I wish the kebele [administration] could issue me the poverty certificate. That would have helped me get the injection for half the price. However, even to get the poverty certificate, I need money to bribe them. ... Although they say treatments are free upon presentation of a certificate, there is still some money we should pay. Forget 25 or 50 birr [$1 or 2], for the poor, even raising 1 birr is tough.* ID09, male caregiver, disengaged.

The health professionals also emphasised the ethical dilemma they face as the result of a restrictive quota which only allows 100 people per primary care facility to receive the fee waiver despite a greater need*.* Those engaged in treatment and their caregivers also expressed their concerns about their ongoing ability to pay as the illness requires long-term access to medications.

*…I have repeatedly gone to the kebele [administration] to get that certificate so I don’t have to pay for his medication every month. Nevertheless, the kebele has been giving me reasons like they do not have a stamp or they need to sit and discuss with the chairman about my request. I am paying now, though I am not sure of how I am going to continue*. ID26, female caregiver, engaged.

Although there was an ongoing struggle to afford treatment, those in treatment appeared to have found ways to raise money or had relatively better social support.

*I live with my mother. She is very old. My brothers and sisters are supporting both of us…I sometimes worry about the cost of treatment…. My two brothers have been assisting me with the money for treatment. I sometimes worry about the future....*ID20, female service user, engaged.

Many of the service users whose situation had improved with the medication referred to treatment as their “*life”*. In addition to the benefits of improved health, the HEWs highlighted the positive value of testimonies of people who have recovered with the medication. Involving these people in raising awareness has facilitated mobilisation of funds by community members.

*The community gets very excited when they hear the news of someone feeling better with medication. That actually encourages them to do anything within their reach including selling their land to pay for treatment. Seeing someone recover is the key driver of seeking treatment.* HEW FGD 2.

Poverty was inextricably linked with social support because of the reciprocity required to draw upon social networks. Participants from poorer households confirmed that social support is not structured around purely altruistic values. The ability to ‘give back’ dictates the type and extent of social support one could access.

*I can’t do anything to have him treated. People these days do not help if you don’t have money to reciprocate. They do not lend you money for treatment. To help with treatment, people check what you have first*. ID16, male caregiver, non-engaged.

The need to conceal poverty, pressure to maintain one’s dignity and unwillingness to disclose poverty also emerged as barriers hindering access to PHC-based mental healthcare. While the majority of the participants admitted their own poverty, disclosing to neighbours and seeking their assistance was not positively perceived as described below:

*There is no one to help us. I am shy myself. I do not want to ask people to help me. People tell me to beg others to help him but I do not want to face people with such a request. I do not want to be seen begging people.* ID08, female caregiver, disengaged**.**

Reluctance to request assistance to avoid burdening others was mentioned to be common among those who disengaged.

*I don’t have money to buy the tablets. I also did not want to bother anyone at this age. That is why I did not go. I do not want to tell the doctors that I don’t have money. This is the first time I told someone that I did not go because I do not have money*. ID04, male service user, disengaged.

### Medication related barriers

Access to medication was a valued and defining feature of PHC-based mental healthcare in the eyes of most respondents, including people with SMD. Only two of the disengaged service users reported that they would prefer traditional care over modern care, citing the curative properties of traditional healing compared to biomedical medicines:

*There is a woman living around the mountain …. Her treatment really eased my illness….. Though my body was weak from the non-stop vomiting, the digimt [bewitchment] left me….The modern medication is not potent enough to induce vomiting…. It induces sleep instead. You won’t feel energetic even when you wake up. When the traditional medicine makes you feel tired, you would at least be happy because you would see something leaving your body.* ID36, Female service user, disengaged.

Some of the disengaged participants remarked that they would combine traditional treatment with modern care to increase effectiveness. On the contrary, another group of engaged service users reported that holy water and tablets should be mutually exclusive as the holy water necessitates people to fast while the medication requires a good diet.

PHC workers reported that health centres had only intermittent supplies of medication. They expressed their fear that a single experience of medication being out of stock may lead a service user to miss subsequent follow-up appointments.

Next to poverty, lack of choice of medication and intolerable side-effects were among the frequently cited reasons for treatment discontinuation. PHC professionals and service users within and outside the care system highlighted that many people discontinue treatment due to medication side-effects:

*There was a man in our village who started treatment at the nearby health centre. We had a report from the health centre that he stopped the treatment. When we went to talk to him about his situation, he said the medication is too strong for him so he decided not to return to the health centre.* HEW FGD 1.

Interviews with service users and their caregivers confirmed that they have little or no opportunity to negotiate their treatment. The power dynamics within the health professional-patient relationship around medication was evident. As a result, settling for a poorer outcome was common.

*It is a huge deal for me that they [affected family members] are sleeping at home after the tablets. It is priceless that they stayed at home. They sleep well and I sleep well too. I won’t fear that the hyena might eat them. They do not go to anyone’s house. Previously they used to walk aimlessly. Now all of them are at home. That is enough for me. I don’t want to go to the doctors with complaints. What if they deny me the tablets? …. How can I tell the doctor that their medication did not work? Who am I to do so?* ID06, female caregiver, disengaged.

Caregivers of those who had discontinued treatment and some of the service providers remarked that the health centres are not fully equipped with the required antipsychotic medications that are effective enough to lead to recovery, particularly long-acting injectable medications. The HEWs also agreed that there are people whose mental health does not improve with the treatment they receive at the health centre:

*We have a woman in our neighbourhood who said she had to stop taking her medication because the medication worsened her situation. The health centre tried to give her another one but still her situation didn’t improve.* HEW, FGD2**.**

Concerns about adequacy of treatment were shared by the majority of the PHC workers. They emphasised that there are very limited medication options, including medications to alleviate side-effects. The majority of service users with such complaints go without any help. The few people who have sufficient resources travel to specialist services in Addis Ababa seeking better medication.

### Problems of long-term illness

Several challenges were reported under this section and are presented as follows.

(a) *Prolonged engagement with care:* Prolonged engagement with care was not regarded positively by service users and their caregivers, both those in and out of treatment. Many of the reasons were related to the chronicity of the problem and the stigma associated with long-term involvement with mental health services. A caregiver who is also a service user shared his experience of stigma as:

*I wish I could stop the treatment myself…I don’t want to continue taking the medication for a long time. People look down on me because I am mentally ill. They stigmatise us for being on this treatment for a long time. They think of us as aliens.* ID11, male caregiver/service user, disengaged.

Some of the caregivers shared their fears regarding prolonged use of medication and the associated side-effects, as highlighted below:

*It makes me really sad. He can’t stop his medication unless the doctors tell him to stop. Taking the medication every day is difficult and there are side-effects like constipation and others. But this is what has happened already. I can’t change his situation if I complain. This is what he has to do for recovery.* ID29, female caregiver, engaged.

(b) *Misunderstanding the course of the illness* *:* Misunderstanding the course of the illness, especially after severe symptoms have subsided, was reported to be an important reason for treatment discontinuation. Some of the participants refused to attend subsequent follow-up consultations, claiming that they had recovered. Respondents described that help-seeking was not initiated between illness episodes because people felt well. Rather, they would wait for the next severe episode to come before looking for treatment. The HEWs reported that some of the service users and caregivers balanced the costs of continuing to pay for medication against the immediate consequences of illness: when the person has improved, economic concerns predominated.

*Some people don’t discontinue treatment fully. Someone in our village stopped taking the medication a while ago when he felt better. He started again when we followed him up and encouraged him. People go to health centres when they have money for medication and stop when they feel better to cut costs*. *Not many people discontinue treatment fully.* HEW FGD2.

Religious healings that aim at cure rather than recovery were appreciated by most of the service users and their caregivers. According to some of the families, people spent over 2000 Birr [$100] and travelled to distant places with the hope of cure.

(c) *Lack of autonomy:* In addition to poverty, service users who had disengaged remarked that they had low levels of autonomy and lacked the necessary information as to when to initiate contact with mental health service providers. This was also shared by potential service users who did not access care:

*I am dependent on my family for my living. I do not know why they decided not to take me to the health centre. My brother knows about the service but I am not sure if he told my parents. My parents should be told about the service for me to be able to access treatment.* ID17, male potential service user, non-engaged**.**

(d) *Lack of tracking mechanism for people with SMD:* The heavy workload of the PHCs and lack of tracking mechanisms for people who discontinued care was mentioned as a barrier by PHC workers and HEWs. PHC workers reported that the health centre adopted effective community-based tracking mechanisms for TB and HIV/AIDS. Nevertheless, they underlined that health centres failed to introduce such interventions for people who discontinued mental health treatment.

(e) *Lack of social support:* The decline of social support over the years was also mentioned as one of the reasons why people discontinue treatment. Social support from neighbours and relatives was mentioned to be higher in the first few months after the illness is recognised but will continue to decline with time. In some cases, caregivers stated that they did not want to request support from others, especially when those people had previously extended some help to the family.

*….Everyone around us is tired of my requests. His friends were with us through the process but now they are also very fatigued. They used to come to our house every day to make sure that he is taking his medication but now they are not doing that anymore. They were consistent for the first six months. Now I do not want to bother them again because they have given what they can*. ID15, female caregiver, non-engaged.

Participants indicated that people outside of their immediate family may give counsel but are remote when providing practical help comes into play.

(f) *Multiple co-morbidities:* All of the interviewees acknowledged that the presence of multiple co-morbidities posed additional challenge to PHC-based mental healthcare over time. Caregivers of people who had other medical conditions such as diabetes, hypertension or cardiac issues tended to focus on the noticeable and most troublesome problem rather than an episodic SMD. Besides, having physical disability and severe functional impairment also caused added burden on caregivers in terms of conveying the person to the health centre.

### The nature of the illness

Some of the caregivers indicated that severity of illness is often accompanied by violence towards others. In our attempts to include service users from the non-engaged and disengaged groups, being too unwell (*n* = 10) and homelessness were common reasons for not participating. Thus, escorting an acutely disturbed person to a PHC was reported to be challenging even after the service has been made locally available.

*Recently we heard that there is treatment at the health centre. We rented a cart to get him there. We had to pay 80 birr. My two sons were trying to help him board. He beat them both and escaped.* ID 15, female caregiver, non-engaged.

The currently available service does not respond to individual requests for assistance when the service user resists. Caregivers of people who discontinued treatment and those who did not access treatment mentioned that the PHCs have little or nothing to do in cases of resistance of the service user.

*They diagnosed him and gave him tablets….He did not cooperate at all. What can we do when he refuses?* ID08, female caregiver, disengaged**.**

Some of the PHC staff members mentioned that they are willing to provide support when they are called by caregivers who are unable to convey their acutely disturbed family members to the PHC. However, they underlined that the current setup of the PHC does not support proactive care, such as conducting community outreach. They added that any PHC staff member who self-initiated community outreach would be considered as taking an unexcused absence from work. According to the PHC staff interviewed, such initiatives could be considered as a conspicuous departure from the PHC’s tradition which only provides facility-based services.

Caregivers in all groups reported that supporting a person with SMD who also has co-morbid alcohol use problems is especially burdensome. Currently, people who are receiving care at PHCs are advised to stay away from alcohol while on medication. Hence, premature discontinuation of medication to use alcohol was reported to be common. A similar attitude was expressed by the majority of those who were on treatment as they wished to discontinue their medication someday so they can drink alcohol without the feeling of guilt.

The table below summarises results under each of the four themes as reported by the engaged, disengaged and non-engaged people with SMD (Table 2).

**Table 2:** Summary of barriers experienced by people with SMD

	Engaged	Disengaged	Non-Engaged
Poverty	Difficulty to maintain ongoing access to treatment (not being able to cover cost of treatment)	Difficulty to maintain ongoing access to treatment	Inability to initiate treatment due to lack of money for direct and indirect costs of treatment
	Difficulty to obtain poverty certificate	Difficulty to obtain poverty certificate	Lack of social support (inability to reciprocate)
		The pressure to maintain dignity	
		Concealing poverty	
Medication-related barriers	Unreliable medication supplies	The belief that holy water and modern medication should be mutually exclusive	
	Medication side-effects	Intolerable medication side-effects	
		Lack of some medications	
		Ineffective medications	
Long-term care	Medication-side-effects from prolonged use	Stigma of long-term engagement with care	Presence of other co-morbid illnesses
	Declining social support over years	Looking for cure	Having physical disability
	Presence of other co-morbid illnesses	Low personal autonomy	Having severe functional impairment
		Presence of other co-morbid illnesses	
The nature of SMD	The challenge from co-morbid alcohol use	The challenge from co-morbid alcohol use	The challenge from co-morbid alcohol use
	Wish to discontinue treatment to use alcohol (although engaged)	Lack of help for families when service users are uncooperative	Severity of the illness
	Spontaneous improvement	Premature discontinuation of medication to use alcohol	Not wanting treatment
			Violence towards others

The above comparison of the engaged versus the non-engaged does not show a substantial difference in terms of their socioeconomic status or coping. A very small proportion of the engaged people were able to draw upon their social support although it was not expected to be ongoing. It was also reported that some of the engaged managed to stay in care because their health professionals paid for them.

## Discussion

Previous studies exploring equitable access to mental healthcare were largely conducted in the absence of biomedical services. The implementation of locally available mental healthcare through PRIME provided a platform for understanding the practical barriers to available mental healthcare (in the primary care setting) in this rural, low-income country setting. We were able to explore the spectrum of engagement with care, including those who had never engaged with care, those who engaged initially and then disengaged or those who were engaged throughout. Ongoing engagement with care was found to be challenging for all groups of respondents, even those who were engaged currently. Although the provision of more locally available care had certainly improved accessibility of care, major challenges remained. The main barriers to ongoing engagement were poverty, unreliable availability of appropriate medications, the demands of a long-term illness and the very nature of SMD.

### Poverty

There is established evidence regarding the multiple inter-linkages between poverty and mental illness [23, 24]: SMD leads to disability and social exclusion for the person and their family. Reduced earning potential and declining productivity of the individual and their caregivers often leads to poverty [25]. Expenditure on help-seeking can be catastrophic and compound the impoverishment caused by the illness [25]. At the time of the study, most payment for healthcare in Ethiopia was out-of-pocket and borne by the family, although some participants in the study had been able to benefit from a fee waiver system for the ‘poorest of the poor’. Initiatives to introduce community-based health insurance are underway and have the potential to alleviate some of the direct costs of care [26]. However, the impoverished and marginalised status of families who have a person with SMD may yet undermine access [27]. Supplementing the existing fee waiver system with cash transfers could facilitate utilisation of services [28, 29]. Beyond this, providing livelihood support and strengthening the newly introduced community-based insurance system [30] will reduce household level vulnerability [31] and promote more complete recovery in persons with SMD [32].

The identified linkages between poverty, social support and access to care are consistent with the findings from our formative qualitative study [33]. Social support was reported to be crucial for ongoing access to care, but liable to decrease over the years, mainly attributable to the limited ability of families to engage in reciprocal relationships with their community. In this sense, social support is perceived as an obligation rather than a free gift. In a study of social capital in rural Ethiopia, having high social capital was a stressor rather than being a protective factor for depression symptoms [34]. Initiatives to retain people with SMD in care cannot assume that families will have support networks to draw upon or, indeed, that the community will have the capacity to provide support on an ongoing basis, raising the potential need for state intervention for selected cases.

### Medication

All participants in the study spoke of the centrality of medication to care. Ensuring a sustainable supply of medications goes beyond ensuring availability of medication, also extending to include mental health policies and budget allocation [35]. Strengthening procurement channels is a prerequisite. Moreover, guaranteeing affordability of medications is key to ensuring continuing access to care and reducing rates of treatment discontinuation [36].

Consistent with findings from other LMICs [36–38], procurement only of older generation medications with burdensome side-effects, and having only one medication per drug category were reported as limitations. This lack of choice undermined person-centred approaches whereby treatment plans are uniquely tailored to the specific needs of each individual. The lack of familiarity with other treatment options also indicates low levels of empowerment of service users, exacerbated by low expectations of care more generally [39]. Community-based rehabilitation approaches are being trialled in the same population and have potential to enhance the capacity of service users to negotiate their treatment, at least with respect to dosage and appropriateness of medications [40].

In our formative study, participants anticipated that people would rely on traditional and faith healings and that biomedical care would have low acceptability [41]. The current findings indicate that the benefit from medication was widely acknowledged by both service users and caregivers. Contrary to what was reported by another implementation research in Tanzania [42] and our previous study [9], only two people preferred traditional treatment to PHC based biomedical care, although many participants continued to use traditional approaches alongside biomedical treatment. Similar to a study from Haiti [43], in our study the relevance of barriers from culturally influenced explanatory models appeared limited in practice. In both Haiti and our study, there was a difference in perspectives between service users and service providers, with service providers placing more emphasis on the influence of culture and explanatory models. In our study, pragmatism driven by observed benefits of medication appeared to have been the dominant factor influencing help-seeking for service-users.

### Problems of long-term illness

Although people were motivated by positive treatment outcomes, such as reduction in severe symptoms and improved functioning, prolonged engagement with care was not positively perceived. The need for health system change was apparent, particularly in making sure that service users are aware and are able to make informed decisions about their health. The innovative care for chronic conditions framework (ICCCF) works to maximize the flexibility of health systems to provide better care for long-term health challenges [44]. The application of such a framework in low-resource contexts has potential to enhance proactivity of the system [33, 45].

The power differential between service users and PHC workers affects the discourse of the helping relationship [46]. Low service user awareness about the need for chronic care and limited understanding of medication side-effects and mechanisms to overcome them were major challenges discouraging prolonged engagement with care. A study conducted in the same setting reported that mental health service users and caregivers are disempowered due to the longstanding marginalisation [47]. Therefore, expectations from healthcare providers were limited to prescription of medications only. Empowering service users to negotiate their own treatment, and equipping them with skills such as managing their illness through life-style changes could promote better mental health. In a recent systematic review of studies from LMICs [48], there were reports of isolated initiatives to engage mental health service users in advocacy, but limited evidence on effective ways to increase their involvement in care and system processes. Work is ongoing in the study site to develop and evaluate models of enhancing service user involvement [49].

Integration of mental health and physical health interventions is necessary to reduce the high rates of mortality [50]. Introduction of innovative and proactive healthcare system changes that support community outreach services and forging partnerships with service users could be essential in addressing the current barriers [45].

### Nature of illness and other barriers

Access to mental healthcare for people with SMD has additional complexity compared to most physical health disorders due to the very nature of the illness. Many people with SMD do not recognise the illness and fail to initiate help-seeking. In this study, we approached 10 people with SMD who had not engaged with care, all of whom were acutely disturbed and some reported to be missing. Some caregivers of non-engaged people with SMD reported that they could not escort their family members to PHCs due to the severity of the illness. This indicates failure of the existing model of care to address the ‘inverse care law’ [51], whereby the people who most need care are least likely to be able to access care. The introduction of ambulatory mental health services and crisis intervention would facilitate access by people who are unwell or those who are too difficult to convey. The UN convention on the rights of persons with disabilities (CRPD) targets all forms of discrimination against people with physical and mental disabilities by stating the right to liberty, including protection from involuntary treatment [52]. However, for people with SMD who have severely impaired decision-making capacity, application of such frameworks may easily translate into not receiving treatment at all in a setting like rural Ethiopia, with all of the attendant risks of human rights abuses [53]. It is important to have a balanced interpretation and application of CRPD, such that CRPD would support the development of legal frameworks that enable people without capacity to consent to access care while protecting their autonomy [54]. Currently, there is lack of legal framework applicable to the Ethiopian context.

**Fig. 1:**
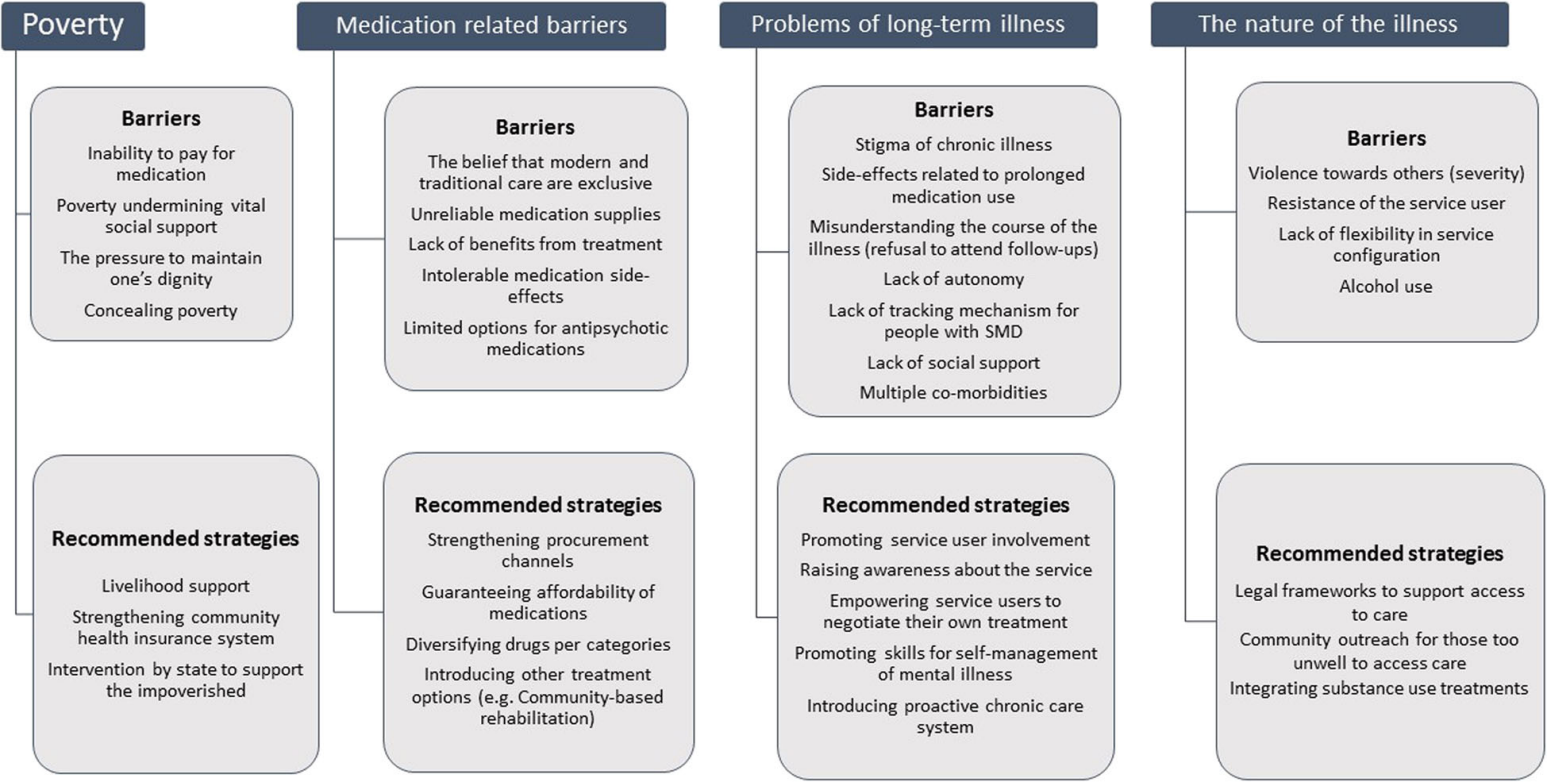
Conceptual model

The conceptual model below summarises the barriers under each of the themes and the recommended strategies based on the findings of this study (Fig. 1).

### Strengths and limitations

One of the strengths of this study is that it involved the voices of people with SMD who are non-engaged with care and their caregivers, an important group whose perspectives are often missed. The study also viewed engagement as a continuum and interviewed service users and carers with various degrees and stages of engagement. Nevertheless, only three non-engaged people with SMD were interviewed because of the difficulty of finding a potential service user eligible for the interview, due to their acute illness. Another important limitation of this study is that we had to exclude some people (*n* = 5) for not speaking Amharic. Power differences between the interviewers and the study participants might have resulted in social desirability bias, although we tried to minimise this by conducting the interviews in the natural settings of the study participants and making every effort to convey to respondents that we valued their opinions and experiences.

## Conclusion

Economic interventions may be needed to support ongoing engagement with care for people with SMD. Systems of care for chronic illness need to be strengthened in combination with legal frameworks. More flexible service configurations are likely to be necessary, incorporating elements of the chronic care framework, with particular emphasis on service user empowerment. Expanded options for adequate, affordable and effective medication and psychosocial interventions are required for person-centred care.

## Additional file

Additional file 1: Codebook. (XLSX 44 kb)

## Abbreviations

HEW: Health Extension Workers
LMIC: Low- and Middle-Income Countries
PHC: Primary Health Care
PRIME: PRogramme for Improving Mental health carE
SMD: Severe Mental Disorders
